# Migration Degree of Selected Mycotoxins in the Distillation Process and Their Determination in Distilled Spirits from Pilot-Scale Continuous Distillation

**DOI:** 10.3390/foods12234189

**Published:** 2023-11-21

**Authors:** Jung-Ah Shin, Ki-Teak Lee

**Affiliations:** 1 Department of Marine Bio Food Science, Gangneung-Wonju National University, 7 Jukheon-gil, Gangneung 25457, Republic of Korea; jashin@gwnu.ac.kr; 2 Department of Food Science and Technology, Chungnam National University, 99 Daehak-ro, Yuseong-gu, Daejeon 34134, Republic of Korea

**Keywords:** mycotoxin residue, distillate migration, distillation, ethanol fermentation, distilled spirits

## Abstract

Mycotoxins (ochratoxin A (20 ppb), aflatoxin B1 (40 ppb), deoxynivalenol (4 ppm), and zearalenone (800 ppb)) were intentionally added to rice bran raw materials. After fermentation, their contents were determined in the distillate and distillery stillage obtained using single-stage and continuous pilot plant-scale columns. After single-stage distillation, aflatoxin B1, deoxynivalenol, and zearalenone were not detected in the distillate, indicating that even if a certain amount (four times the maximum residue limit (MRL)) was present in the raw material, it would not remain in the distillate after fermentation and distillation. Most mycotoxins remained in the distillery stillage, and their residual rates ranged from 54.0–96.2%. For ochratoxin A, 0.19 ppb was found in the distillate and this migration occurred in three consecutive distillations (0.11–0.22 ppb). Ochratoxin A and aflatoxin B1 were not detected in the distillate (alcohol content 93.9% and 95.4%, respectively) obtained from the contaminated fermented liquid (approximately three times the MRL based on the raw material) using the pilot-plant scale continuous distillation column. Therefore, the migration of mycotoxins is difficult when the distilled spirit is produced using a continuous distillation column, even if the raw material is contaminated with certain amounts of the investigated mycotoxins.

## 1. Introduction

Distilled spirits are generally obtained by fermenting grains (mainly rice, sweet potatoes, and tapioca) as raw materials, followed by distillation. The distilled spirits are neutral alcohols composed of approximately 95% ethanol and 5% water which is their azeotropic ratio (95.6:4.4%) [1]. If distillation does not proceed properly, a considerably small amount of byproducts from fermentation can exist in the distilled spirit, in which methanol and higher alcohols (i.e., fusel oils) such as propanol, isobutanol, and amyl alcohols are typical impurities [2]. Therefore, the distillation process is an important factor in maintaining the high purity of the distilled spirit, resulting in a colorless and transparent appearance with no suspended matter or off-flavor. The distillation process can effectively reduce contamination from mycotoxins, which are easily produced in grains and can pose considerable health risks.

Mycotoxins are potentially hazardous secondary metabolites produced by naturally occurring molds (*Aspergillus*, *Penicillium*, *Fusarium*, etc.) during the storage and distribution of agricultural products [3]. Mycotoxins that often occur in grains, which are raw materials for alcohol production, include aflatoxin B1, ochratoxin A, deoxynivalenol, and zearalenone. These four mycotoxins are particularly controlled by the Korean Ministry of Food and Drug Safety (KMFDS) with national standard specifications. Furthermore, the International Agency for Research on Cancer (IARC) classifies aflatoxin B1 as a Group 1 and ochratoxin A as a Group 2B human carcinogens [4,5]. Aflatoxin B1 is strictly regulated because it is highly carcinogenic. In South Korea, aflatoxin B1 is regulated to amounts less than 10 ppb in grains, beans, nuts and their simple processed products, and less than 50 ppb in feed. In Japan, it must not be detectable in food, and the acceptable standard in feed is 10 to 20 ppb. In the United States, Brazil nuts, peanuts, their processed products, and pistachios are regulated to less than 20 ppb, and feed is regulated to less than 20 to 30 ppb [6]. Grains that have been stored for a long time are at risk of mycotoxin contamination. Therefore, if the raw material is contaminated with mycotoxins, whether or not they remain in the distilled spirit is an important factor in assuring safety. Mycotoxins can be removed to different degrees by physical processing, such as dehulling, milling, and heating, and by chemical processing, such as washing through an alkali treatment (i.e., sodium carbonate solution). As alcohol goes through the steaming, fermentation, and distillation processes, the raw ingredients do not remain in the final product. Even if raw materials are contaminated with mycotoxins, whether or not they remain will be determined depending on the physical and chemical properties of the mycotoxins. In particular, deoxynivalenol, which has a high solubility in water (log *p* = −0.71, Table 1), can be removed by up to 72–75% using an alkali washing step [7]. Also, aflatoxin B1 is unstable under acidic or alkaline conditions and tends to degrade almost completely at temperatures above 160 °C [7,8,9], whereas ochratoxin A is stable up to 180 °C and undergoes partial decomposition under normal cooking conditions [8,9,10]. Meanwhile, deoxynivalenol exhibits 16–100% degradation at 125–250 °C and 30–66% degradation in aqueous solutions with a pH of 1–3, whereas zearalenone, an endocrine disruptor with estrogenic activity, has high thermal stability at a neutral pH [10,11]. At pH 10, deoxynivalenol starts to partially decompose after 60 min at 100 °C, and the decomposition of zearalenone is faster in alkaline conditions than under neutral or acidic conditions [10,12]. Therefore, heat (temperature and time) and pH are important factors affecting mycotoxin decomposition. In the case of beer brewing, Inoue et al. [13] reported that the contents of aflatoxin, ochratoxin A, and zearalenone were reduced to less than 20% of their initial concentrations through mashing, filtration, boiling, and fermentation processes. Mycotoxins such as these in distilled spirits can be removed by decomposition and adsorption. The reduction in mycotoxins is mainly caused by adsorption to the grains used, except zearalenone, which is metabolized into less toxic compounds during the fermentation process; therefore, mycotoxins naturally disappear during the brewing process.

Previously, we reported that substances (e.g., pesticides) could migrate to the distillate depending on the distillation method and conditions [14]. In the case of mycotoxins, barley soju obtained through a small-scale distillation process was studied, in which mycotoxins did not migrate into the barley soju when distilled at an atmospheric pressure and at a low pressure (100 mmHg) [15]. To the best of our knowledge, it is difficult to conduct a pilot-scale study of mycotoxin migration into distilled spirits. In this study, raw material (i.e., brown rice) contaminated with four types of mycotoxins (aflatoxin B1, ochratoxin A, deoxynivalenol, and zearalenone) was distilled and fermented, and each distillate obtained through single-stage and pilot plant-scale continuous column distillation was investigated to determine whether mycotoxins could be detected, and therefore, the degree of migration.

## 2. Materials and Methods

### 2.1. Materials

Rice bran powder, a commercial-grade coenzyme, liquefying enzyme solution, diastatic enzyme solution, and commercial distilled spirit were provided by the Korea Alcohol and Liquor Industry Association (KALIA). Aflatoxin B1 (CRM46323), ochratoxin A (34037), deoxynivalenol (D0156), and zearalenone (Z2125) were purchased from Sigma-Aldrich Corp. (St. Louis, MO, USA). Aflatoxin B1 and ochratoxin A were prepared in benzene and acetonitrile (98:2, *v*/*v*), and 100% acetonitrile, respectively. Deoxynivalenol and zearalenone were provided in powder form and dissolved in ethyl acetate and methanol (95:5, *v*/*v*) and 100% acetonitrile, respectively.

### 2.2. Sample Preparation of Mycotoxin Contamination for Ethanol Fermentation

**Figure 1:**
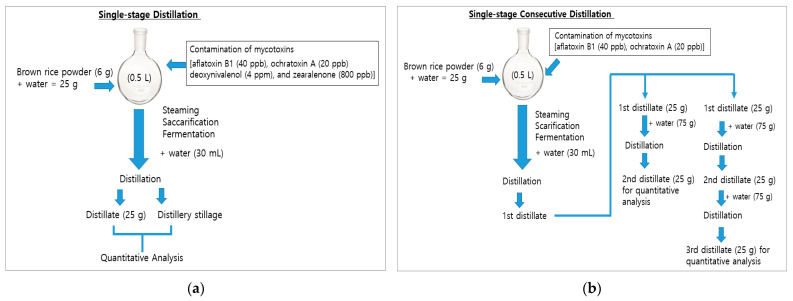
Single-stage distillation (**a**) and single-stage consecutive distillation (**b**).

Rice bran powder (6 g) was placed into a 500-mL round flask. All mycotoxin solutions (aflatoxin B1, ochratoxin A, deoxynivalenol, and zearalenone) for contamination were diluted in distilled spirit to a corresponding concentration. This aimed to be four times the maximum residue level of mycotoxins permitted by the Korean Ministry of Food and Drug Safety (MFDS) in brown rice (Figure 1).

The steaming process was initiated by adding 0.22 mg of ammonium sulfate and distilled water to adjust the total weight of the sample (25 g), and 2.8 µL of the liquefying enzyme solution at 85 °C for 20 min followed by heating at 95 °C for 1 h. Then, the temperature of the mixture was lowered to 70 °C and maintained for 30 min; thereafter, 22 µL of the diastatic enzyme solution, 2.2 mg of the coenzyme, and the mixture were heated at 70 °C for 1 h. After cooling to 33 °C, 2.2 mL of the prepared *Saccharomyces cerevisiae* (Jenico Instant yeast 1 g/5 mL, activated at 37 °C for 20 min) was added and fermentation was conducted at 32 °C for 4 d. During the fermentation process, the round flask was closed with a cork. The weights of each flask were measured to confirm that fermentation was performed without any abnormalities. After the completion of fermentation, 23.9–24.3 g of fermented liquid was obtained. The study flow of sample preparation is shown in Figure 1a.

### 2.3. Preparation of Distillate and Distillery Stillage from Single-Stage Distillation

Single-stage distillation was performed with the 500-mL round flask using a rotary vacuum evaporator (EYELA, Rotary Evaporator N-1000, Digital Water bath SB-1000, Aspirator A-3S, Rikakikai Co. Ltd., Tokyo, Japan). Single-stage distillation was conducted while maintaining a temperature of 80 °C, pressures of 50–70 cmHg, and a condenser temperature of −10 °C. To obtain approximately 25 g of distillate, 30 mL of additional distilled water was added to the 500-mL round flask during the distillation process. Subsequently, the distillate and all flasks with remaining distillery stillage were sent to the Korea Advanced Food Research Institute (KAFRI). KAFRI is a specialized analysis institute certified by the Ministry of Food and Drug Safety of Korea. After obtaining the mycotoxin content in the samples, the distillate migration rates (DMRs) and distillery stillage residual rates (DSRRs) were calculated as follows: DMR (%) = (content detected in the distillate/content detected in the rice bran powder after spiking) × 100; DSRR (%) = (content detected in the distillery stillage/content detected in the rice bran powder after spiking) × 100. The aflatoxin B1 and ochratoxin A contents in the distillate were obtained (Figure 1a,b). The distillation conditions and sample treatments were as described above.

### 2.4. Preparation of Distillate Fractions for Determining Aflatoxin B1 and Ochratoxin A from Pilot-Plant Scale Continuous Distillation Column

Korean distilled spirit manufacturing companies primarily use distillation columns. This method was used for distillation at the pilot-plant scale. Using a distillation column, distillation was performed after 1500 g of the feed mixture (250 g of fermented liquid + 1250 g of 5% ethanol solution) was intentionally contaminated with aflatoxin B1 (2 ppb) and ochratoxin A (3.3 ppb). The alcohol content of fermented liquid was 12% (*v*/*v*). The contaminated feed mixture was placed in a distillation vessel maintained at 104.5–105.5 °C. The temperature at the top of the distillation column (stainless steel) was not controlled until the intended volume of the distillate fraction was obtained. The condenser temperature was maintained at 50 °C. The distillation column, 25 cm in diameter and 200 cm in total height, consisted of five sections in which copper chips (0.25 inch) were irregularly filled in each section. During distillation, three and five fractions were obtained from the feed mixtures contaminated with ochratoxin A and aflatoxin B1, respectively. After measuring the alcohol percentage using a density meter (DMA 4500 m, Anton Paar GmbH, Graz, Austria), the distillate fractions were analyzed at KAFRI to determine the mycotoxin content.

### 2.5. Preparation of Distilled Spirits and Distillery Stillage for Determining Aflatoxin B1 and Ochratoxin A Contents from Pilot-Plant Scale Distillation Column

Fermented liquid (1950 g) obtained from the manufacturer was intentionally contaminated with aflatoxin B1 (7.69 ppb) and ochratoxin A (3.84 ppb); each corresponding concentration was aimed to be three times the maximum residue level of mycotoxins allowed in brown rice. All procedures, as described above, were performed according to the continuous distillation method except that the temperature (77.8–78.0 °C) was maintained at the top of the distillation column. After distillation, the distillate (155 mL) was obtained as a distilled spirit. The contaminated fermented liquid, distilled spirit, and distillery stillage were analyzed at KAFRI to examine the contents of aflatoxin B1 and ochratoxin A.

### 2.6. Analysis of Ochratoxin A

These procedures were performed according to the Korean Food Code [16]. A sample (approximately 20 g) was weighed, and 100 mL of a solution of acetonitrile and water (6:4, *v*/*v*) was added. The mixture was then homogenized for 5 min and filtered through a Whatman No.4 paper. The filtrate (5 mL) was mixed with the prepared phosphate-buffered saline solution (55 mL) and passed through a Whatman GF/A glass microfiber filter. Subsequently, the filtrate was placed into an immunoaffinity column (Ochra Test™, VICAM, Milford, MA, USA) and eluted with water. The residue in the column was removed using a vacuum pump and eluted with methanol. The eluate was concentrated with nitrogen gas at 50 °C, re-dissolved in 1 mL of acetonitrile: water: acetic acid (49.5:49.5:1, *v*/*v*/*v*) and filtered through a 0.2-μm membrane filter for analysis. A calibration curve was prepared using 60% acetonitrile in 1% acetic acid solution with 1, 2.5, 5, 10, and 25 ppb of ochratoxin A standard. A Shiseido Nanospace SI-2 system (Osaka Soda Co., Ltd., Osaka, Japan) was used for ultraviolet detection at a wavelength of 220 nm. Shiseido Capcell Pak C18 (4.6 × 250 mm, 5 μm, Osaka Soda Co., Ltd., Osaka, Japan) was used. Separation was achieved using an isocratic elution consisting of 60% acetonitrile in 1% acetic acid solution at a flow rate of 1 mL/min. A Shiseido Nanospace SI-2 system (Osaka Soda Co., Ltd., Osaka, Japan) was used for fluorescence detection (Ex 333 nm, Em 460 nm). A Shiseido Capcell Pak C18 (4.6 × 250 mm, 5 μm, Osaka Soda Co., Ltd., Osaka, Japan) was used. Separation was achieved via isocratic elution with 60% acetonitrile in 1% acetic acid solution at a flow rate of 1 mL/min.

### 2.7. Analysis of Aflatoxin B1

The procedures were performed according to the Korean Food Code [17]. Samples (approximately 20 g) were weighed, and 100 mL of a solution of acetonitrile and water (7:3, *v*/*v*) containing 1% NaCl was added. The mixture was then homogenized for 5 min and filtered through a Whatman No.4 paper. The filtrate (10 mL) was mixed with 1% Tween 20 solution (30 mL) and filtered using a Whatman GF/A glass microfiber filter. Subsequently, the filtrate (20 mL) was placed in an immunoaffinity column (Afla Test™, VICAM, Milford, MA, USA) and eluted with 10 mL of water. The residue in the column was removed using a vacuum pump and eluted with 3 mL of acetonitrile. The eluate was concentrated with nitrogen gas at 50 °C, and 0.2 mL of trifluoroacetic acid was then added for derivatization (15 min) in the dark. Subsequently, 0.8 mL of an acetonitrile and water solution (20:80, *v*/*v*) was mixed with the eluate and filtered through a 0.45-μm membrane filter for analysis. The calibration curve was prepared using acetonitrile at 1, 2, 5, and 25 ppb of aflatoxin B1 standard after derivatization, as described above. An Agilent 1260 Infinity II system (Agilent Technologies, Inc., Santa Clara, CA, USA) was used for fluorescence detection (Ex 360 nm, Em 450 nm). A Shiseido Capcell Pak C18 (4.6 × 250 mm, 5 μm, Osaka Soda Co., Ltd.) was used. Separation was achieved via gradient elution with 10% acetonitrile (A) and 90% acetonitrile (B) administered at a flow rate of 1 mL/min using the following elution protocol (elapsed time, ratio of solvent B): 0 min, 10% B; 2 min, 10% B; 18 min, 50% B; 19 min, 50% B; 21 min, 10% B; 25 min, 10% B.

### 2.8. Analysis of Deoxynivalenol

This study was conducted in accordance with the Korean Food Code [18]. The sample (approximately 20 g) was weighed, and 100 mL of water was added. The mixture was then homogenized for 5 min and centrifuged (10,000× *g*, 10 min), and the supernatant was filtered through a Whatman GF/A glass microfiber filter. The filtrate (2 mL) was placed into an immunoaffinity column (DON Test™, VICAM, Milford, MA, USA) and eluted with 5 mL of water. The residue in the column was removed using a vacuum pump and then eluted with 3 mL of acetonitrile. The eluate was concentrated with nitrogen gas at 50 °C, re-dissolved in 1 mL of acetonitrile and water (17:83, *v*/*v*), and filtered through a 0.45-μm membrane filter for analysis. A calibration curve was prepared using acetonitrile and water (17:83, *v*/*v*) with 0.1, 0.2, 0.5, 1, and 2 ppm of the deoxynivalenol standard. A Shiseido Nanospace SI-2 system (Osaka Soda Co., Ltd.) was used for ultraviolet detection at a wavelength of 220 nm. A Shiseido Capcell Pak C18 (4.6 × 250 mm, 5 μm, Osaka Soda Co., Ltd.) was used. Separation was achieved via isocratic elution with acetonitrile and water (17:83, *v*/*v*) at a flow rate of 1 mL/min.

### 2.9. Analysis of Zearalenone

This study was conducted in accordance with the Korean Food Code [19]. Samples (approximately 20 g) were weighed, and NaCl (2 g), 1 mL of Tween 20, and 100 mL of 75% acetonitrile were mixed, homogenized for 5 min, and filtered through Whatman No. 4. The filtrate (10 mL) was then mixed with 40 mL of water, followed by centrifugation (5000× *g*, 10 min). The supernatant was filtered with Whatman GF/A glass microfiber filter, and the filtrate (25 mL) was placed into an immunoaffinity column (Zearala Test™, VICAM, Milford, MA, USA) and eluted with an additional 20 mL of water. The residue in the column was removed using a vacuum pump and then eluted with 5 mL of methanol. The eluate was concentrated with nitrogen gas at 40 °C, re-dissolved in 1 mL of a mixed solution (acetonitrile: methanol: water, 15:65:20, *v*/*v*/*v*) and filtered through a 0.45-μm membrane filter for analysis. A calibration curve was prepared with 75% acetonitrile using 50, 100, 250, 500, 1000 ppb of zearalenone standard. A Shiseido Nanospace SI-2 system (Osaka Soda Co., Ltd., Osaka, Japan) was used for fluorescence detection (Ex 275 nm Em 450 nm). A Shiseido Capcell Pak C18 (4.6 × 250 mm, 5 μm, Osaka Soda Co., Ltd., Osaka, Japan) was used. Separation was achieved via isocratic elution with acetonitrile, methanol, and water (15:65:20, *v*/*v*/*v*) at a flow rate of 0.8 mL/min.

## 3. Results and Discussion

### 3.1. Determination of Ochratoxin A, Aflatoxin B1, Deoxynivalenol, and Zearalenone Contents after Single-Stage Distillation

Because CO_2_ is generated during the ethanol fermentation process, whether fermentation has been properly performed can be determined by measuring the weight of the flask before and after fermentation. Theoretically, 0.49 g of CO_2_ is expected to be produced during ethanol fermentation if 1 g of glucose is used (i.e., C_6_H_12_O_6_ = 2C_2_H_5_OH + H_2_O + 2CO_2_). In this study, the weight differences of each flask were 2.53–3.12 g, suggesting that the effect of the applied amounts of mycotoxins on the fermentation was not significant. The weight of all prepared samples was approximately 25 g, making it easy to compare the changes in mycotoxin concentrations for each process. The weights of raw material (6 g of brown rice powder) and distillery stillage were adjusted to 25 g of water, and the distillate was collected by marking the volume corresponding to 25 g on the flask in advance (Figure 1a).

After fermenting the brown rice powder contaminated with ochratoxin A and aflatoxin B1, the amounts detected in the distillate and distillery stillage obtained from a single distillation are shown in Table 2. Aflatoxin B1 was not detected in the brown rice powder used as raw material, whereas the detected ochratoxin A content (maximum residue limits (MRL) = 5 ppb) was 0.32 ppb, which is equivalent to 6.4% of the grain allowance [20]. After single distillation, 0.19 ppb of ochratoxin A was detected in the distillate, which is 3.8% of the grain allowance residue, whereas aflatoxin B1 was not detected. In contrast, 21.6 ppb of aflatoxin B1 and 14.5 ppb of ochratoxin A were found in the distillery stillage. Thus, the migration rate of ochratoxin A was 0.95%, and the residual rate was 72.5%, whereas the migration and residual rates of aflatoxin B1 were 0% and 54%, respectively. Meanwhile, deoxynivalenol was not detected in the brown rice powder; however, 17.43 ppb of zearalenone (MRL = 200 ppb) was detected, which is 8.7% of the allowable amount in grains. In the distillate, however, both deoxynivalenol and zearalenone were not detected, and the residual rates were 76.5% and 96.2%, respectively (Table 2).

From the results, aflatoxin B1 had a lower migration and residual rates (54%) than those of other fungal toxins (73.5–96.2%). In particular, 0.19 ppb of ochratoxin A was detected in the distillate. Although a comparison of heat degradation under the same conditions (temperature, pH, time, etc.) was not performed in this study, aflatoxin B1 was most easily degraded by heat (Table 2). According to Raters and Matissek (2008) [21], when the pure form of aflatoxin B1 was exposed to 150 °C for 60 min, it showed a degradation of more than 70%, whereas ochratoxin A degraded less than 20% at a higher temperature of 180 °C, and this degradation tended to increase when other substances, such as protein and polyphenol compounds, were present together. In contrast, deoxynivalenol in an aqueous solution was degraded by approximately 16% by heat at 125 °C for 60 min, and this degradation tended to increase as the pH decreased [11]. Zearalenone was degraded less than 20% when exposed to 125 °C for 60 min under pH 4, and less than 10% at pH 7 [12]. Based on the results of these previous studies, we believe that aflatoxin B1 was destroyed more by heat during the distillation process than other fungal toxins, resulting in a lower DSRR (Table 2).

One previous study reported a reduction in fungal toxins during the beer manufacturing process [22], in which 14 fungal toxins were artificially spiked into malt and then brewed. Their results showed that less than 20% residual ratios of ochratoxin A, aflatoxin B1, and zearalenone were obtained in the final product (i.e., beer), and such fungal toxins with high hydrophobicity (i.e., with a large log P) were believed to be adsorbed into certain compounds (e.g., proteins) in the filtered spent grains. Among the fungal toxins studied in this experiment, aflatoxin B1 and zearalenone in distillate were not detected with residual rates of 54% and 96.2%, respectively (Table 2). Therefore, toxins are considered to remain in the distillery stillage through adsorption to the solid phase of the fermentation broth (e.g., protein of fermentation product), rather than through migration to the distillate during the distillation process. In addition, zearalenone was less degraded by heat during the distillation than other fungal toxins. In a previous study [22], deoxynivalenol was preferentially present in worts, and was less present than ochratoxin A, aflatoxin B1, or zearalenone in the used grains because it had degraded or was removed during yeast fermentation. In this study, deoxynivalenol, which was thought to have been present mainly in the liquid phase (i.e., water) of the distillery stillage because of its relatively higher hydrophilicity than other fungal toxins, was not detected in the distillate. Consequently, assuming that a certain amount of aflatoxin B1, deoxynivalenol, and zearalenone contaminated the raw materials of brown rice powder, it was not possible for them to remain in the distillate obtained after single-stage distillation. However, although ochratoxin A had the highest log P (5.19) and the largest molecular weight among the fungal toxins used in this experiment, it was likely to remain in the distillate in considerably small amounts under single-stage distillation (Table 1). In addition, the residual ratio of aflatoxin B1 was lower than that of other mycotoxins, which was caused through heat destruction during distillation, owing to its relatively low stability [13].

### 3.2. Determination of Ochratoxin A and Aflatoxin B1 after Repeated Single-Stage Distillation

Only small amounts of ochratoxin A were detected in the distilled water (Table 2). To confirm this migration, re-distillation (2nd and 3rd distillations in Figure 1b) was performed for ochratoxin A along with aflatoxin B1, which was not detected in the distillate, to determine whether ochratoxin A had migrated to the distillate even after repeated single-stage distillation.

Aflatoxin B1, which was not detected in the 1st distillation, was also not detected in the consecutive 2nd and 3rd re-distillations. However, ochratoxin A, which was detected in small amounts in the 1st distillate, was also detected as trace (0.22 ppb) in the 2nd and 3rd re-distillations. Therefore, small amounts of ochratoxin A were found to migrate to the distillate (approximately 13 *v*/*v*% alcohol) under this single-stage distillation condition, in which ethanol was distilled with a considerable amount of water (Table 2 and Table 3). It is unclear as to why only ochratoxin A was detected. However, this might be because the limit of detection (LOD) of ochratoxin A (0.05 ppb) is lower than that of the other toxins (0.18–70 ppb) (Table 4). If so, other toxins may also be present in negligible amounts below the LOD in the distillate.

### 3.3. Determination of Ochratoxin A and Aflatoxin B1 Contents in the Distillate Fractions Using Pilot-Plant Scale Distillation Column

**Figure 2:**
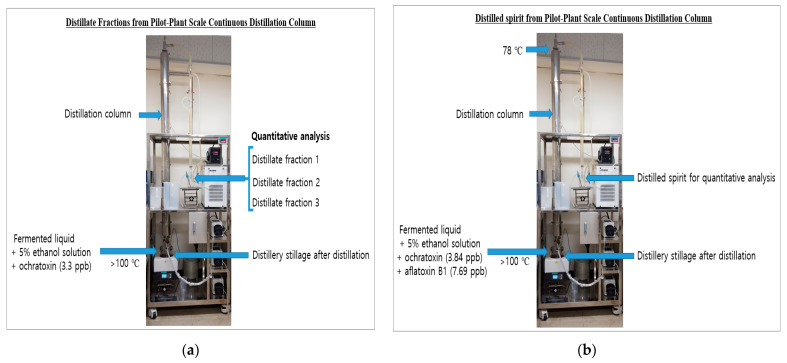
Distillate fractions from pilot-plant scale continuous distillation column (**a**) and distilled spirit from pilot-plant scale continuous distillation column (**b**).

To determine the migration of ochratoxin A according to the alcohol content of the distillate, distillate fractions were obtained using a continuous distillation column (Figure 2a). If the sample was composed of 1250 g of 5% ethanol solution with a small amount of the fermented liquid (250 g, alcohol content = 12%), it would have a high alcohol content in the fraction obtained first; however, in the later fraction, the alcohol content would be gradually reduced when the continuous column distiller was used. Accordingly, the upper temperature of the continuous distillers was allowed to be higher than 80 °C (not controlled), and the fraction of the distillate with a low alcohol content was obtained to measure the contents of ochratoxin A and aflatoxin B1. In all of the distilled fractions obtained by lowering the alcohol content from 87.3% to 33.7%, ochratoxin A was not detected (Table 5). However, 2.35 ppb of aflatoxin B1 was found in fraction 5 (i.e., a distillate in which water was almost distilled) with a reduced alcohol content of 3.3% (Table 6), indicating the possibility that aflatoxin B1 could migrate into a distillate with a considerably low alcohol content. Although a fraction of less than 33.7% was not obtained with ochratoxin A, inferring from the results in Table 6, ochratoxin A, aflatoxin B1, and other fungal toxins are likely to be detected in distillates with considerably low alcohol contents. This phenomenon can be explained by entrainment, in which a less volatile material such as aflatoxin B1 is entrapped in the flowing vapor of water during distillation [23].

Ochratoxin A was detected in the distillate and even in distillates that were re-distilled three times with single-stage distillation, but not in the fractions containing 33.7% or more alcohol (Table 2, Table 3, Table 4, Table 5 and Table 6). Aflatoxin B1, which was not detected in the single distillation, was detected in fractions of 3.3% alcohol. Therefore, it was further investigated as to whether or not the contaminated ochratoxin A and aflatoxin B1 migrated into the distilled spirit (more than 94% alcohol) under experimental conditions similar to those at the manufacturing site.

### 3.4. Determination of Ochratoxin A and Aflatoxin B1 in the Distilled Spirit from Pilot-Plant Scale Distillation Column

In single-stage distillation, distilled spirits with alcohol contents of approximately 94% cannot be produced. Thus, after preparing distilled spirits using a pilot-scale continuous distillation column, the amounts of ochratoxin A and aflatoxin B1 were measured to determine whether they had migrated into the distilled spirit.

After aflatoxin B1 (7.69 ppb) and ochratoxin A (3.84 ppb) were added to the fermented liquid (1950 g), the amounts detected in the obtained distilled spirit are shown in Table 7. The analyzed concentrations of aflatoxin B1 and ochratoxin A in the contaminated fermented liquefaction were 9.64 ppb and 4.05 ppb, showing recoveries (%) of 105 to 125, respectively. The alcohol content of the obtained distilled spirit was 93.9–95.4%, in which no fungal toxins were detected. In contrast, aflatoxin B1 (3.65 ppb) and ochratoxin A (2.70 ppb) were detected in the distillery stillage, with residual ratios of 47.5% and 70.3%, respectively. In addition, the residual rate of aflatoxin B1 was lower than that of ochratoxin A, which was thought to be due to the relatively low thermal stability of aflatoxin B1 described above. Interestingly, the residual rate of each toxin was not significantly different from that of single-stage distillation (Table 2).

## 4. Conclusions

This study was performed to confirm whether mycotoxins remained in distilled spirits (distillate) and distillery stillage that were fermented and distilled after artificial contamination of rice bran raw materials with mycotoxins. In conclusion, aflatoxin B1, deoxynivalenol, and zearalenone were not detected in the distillate from the single-stage distillation. These results mean that even if an amount matching four times the MRL was present in the raw material, it would not remain in the distillate after fermentation and distillation. To determine the migration of ochratoxin A depending on the alcohol content of the distillate, distillate fractions were obtained using a continuous pilot-scale distillation column. The alcohol content of the first fraction obtained was high (about 87%), with the alcohol content gradually decreasing in subsequent fractions as the continuous column distiller was used. The observed results were that ochratoxin A, aflatoxin B1 and other mycotoxins are likely to be detected in distillate fractions with low alcohol contents. This phenomenon can be explained by entrainment, where less volatile substances, such as aflatoxin B1, are captured in the flowing water vapor during distillation.

Ochratoxin A, which was detected in the distillate from single-stage distillation, was not detected in the continuous distillation process used to produce commercially distilled spirit. The most significant difference between the two distillation methods was the alcohol content of the distillate. The distillate obtained from the single distillation process contained more water during which vapor entrainment may occur. Nevertheless, the migration of mycotoxin is difficult when the distilled spirit (94–95% alcohol content) is produced using a continuous distillation column, even if the raw material is contaminated with a certain amount of the mycotoxins studied in this experiment.

## Figures and Tables

**Table 1:** Physicochemical properties ^1^ of mycotoxins.

Mycotoxin	Solubility in Ethanol	Molecular Weight (g/mol)	Vapor Pressure	Log P
Aflatoxin B1	Soluble in absolute ethanol (Sigma)	312.3	2.65 × 10^−10^ mmHg at 25 °C	1.23 (est)
Ochratoxin A	Soluble in ethanol (10–50 mg/mL)	403.8	1.81 × 10^−15^ mm Hg at 25 °C	5.19
Deoxynivalenol	Soluble to 10 mg/mL in 100% ethanol (Sigma)	296.3	6.8 × 10^−11^ mm Hg at 25 °C	−0.71 (est)
Zearalenone	Soluble to 20 mg/mL in ethanol	318.4	0 mm Hg at 20 °C	3–4 (predicted)

^1^ Obtained from the PubChem-National Library of Medicine (NIH), Toxin and Toxin Target Database (T3DB), National Toxicology Program (NTP), and Sigma-Aldrich (Sigma).

**Table 2:** Contents, distillate migration rate (%, DMR), and distillery stillage residual rate (%, DSRR) of contaminated mycotoxins in the distilled spirits and distillery stillage from the single-stage distillation experiment.

	Mycotoxin			
	Aflatoxin B1	Ochratoxin A	Deoxynivalenol	Zearalenone
Unit	ppb	ppb	ppm	ppb
Spiking amount	40	20	4	800
Raw material	ND ^1^	0.32	ND	17.43
Distillate	ND	0.19	ND	ND
DMR (%) ^2^	0	0.95	0	0
Distillery stillage	21.60	14.50	3.06	769.34
DSRR rate (%) ^3^	54.0	72.5	76.5	96.2

^1^ Not detected. ^2^ DMR (%) = (content detected in the distillate/content detected in the rice bran powder after spiking) × 100. ^3^ DSRR (%) = (content detected in distillery stillage/content detected in rice bran powder after spiking) × 100.

**Table 3:** Concentrations (ppb) of aflatoxin B1 and ochratoxin A in distillate during the single-stage re-distillation experiment.

	1st Distillation ^1^	2nd Distillation ^2^	3rd Distillation ^2^
Aflatoxin B1	ND ^3^	ND	ND
Ochratoxin A	0.19	Trace (0.11) ^4^	0.22

^1^ Values are presented in Table 2. ^2^ Experimental scheme is shown in Figure 1b. ^3^ Not detected. ^4^ Value marked as a trace was compulsively integrated.

**Table 4:** Maximum residue limits (MRL), limit of detection (LOD), limit of quantification (LOQ), and calibration curves for analysis of each mycotoxin.

	Mycotoxin			
	Aflatoxin B1	Ochratoxin A	Deoxynivalenol	Zearalenone
MRL ^1^ (ppb)	10	5	1000	200
LOD	0.18 (μg/kg)	0.05 (μg/kg)	0.07 (mg/kg)	2 (μg/kg)
LOQ	0.55 (μg/kg)	0.15 (μg/kg)	0.23 (mg/kg)	6 (μg/kg)
Calibration curves	Y = 1.9765 × 10^−5^ X (R^2^ = 0.999)	Y = 6.6425 × 10^−5^ X (R^2^ = 0.999)	Y = 9.6468 × 10^−6^ X (R^2^ = 0.999)	Y = 4.6 × 10^−4^ X (R^2^ = 0.999)
Standard concentration range	0.1–2 (μg/mL)	1–25 (ng/mL)	0.1–2 (μg/mL)	50–500 (μg/L)

^1^ Maximum residue limits in grains (Korean Food Standards Code).

**Table 5:** Content (ppb) of ochratoxin A in the distillate fractions from the pilot-plant scale of the distillation column.

	Alcohol (%)	Distillate Volume (mL)	Amount of Ochratoxin A	DMR (%) ^1^
Contaminated content	-	-	3.3 ppb (5 μg)	-
Distillate fraction 1	87.3	38	ND ^2^	0
Distillate fraction 2	74.8	28	ND	0
Distillate fraction 3	33.7	110	ND	0

^1^ DMR (%) = (content detected from the distillate/content of spiked ochratoxin A) × 100. ^2^ Not detected.

**Table 6:** Content (ppb) of aflatoxin B1 in the distillate fractions from the pilot-plant scale of the distillation column.

	Alcohol (%)	Distillate Volume (mL)	Amount of Aflatoxin B1	DMR (%) ^1^
Contaminated content	-	-	2 ppb (3 μg)	-
Distillate fraction 1	87.1	60	ND ^2^	0
Distillate fraction 2	47.6	40	ND	0
Distillate fraction 3	15.4	65	ND	0
Distillate fraction 4	5.5	40	ND	0
Distillate fraction 5	3.3	130	2.35 ppb (0.3 μg)	10

^1^ DMR (%) = (content detected from the distillate/content of spiked aflatoxin B1) × 100. ^2^ Not detected.

**Table 7:** Contents (ppb) of ochratoxin A and aflatoxin B1 in the distilled spirit and distillery stillage from the pilot-plant scale of the distillation column.

		Ochratoxin A	Aflatoxin B1
Contaminated content	-	3.84 ppb (7.5 μg)	7.69 ppb (15 μg)
Fermented liquid	Content of mycotoxin	4.05 ppb (7.9 μg)	9.64 ppb (18.8 μg)
	Recovery (%) ^1^	105.5	125.3
Distilled spirit	Obtained volume (mL)	155	155
	Alcohol (%)	93.9	95.4
	Content of mycotoxin	ND ^4^	ND
	DMR (%) ^2^	0	0
Distillery stillage	Content of mycotoxin	2.70 ppb (5.26 μg)	3.65 ppb (7.12 μg)
	Alcohol (%)	0	0
	DSRR (%) ^3^	70.3	47.5

^1^ Recovery (%) = (content detected from the fermented liquid/spiked content in the fermented liquid) × 100. ^2^ DMR (%) = (content detected in the distillate/content detected in the fermented liquid after spiking) × 100. ^3^ DSRR (%) = (content detected from distillery stillage/content detected from fermented liquid after spiking) × 100. ^4^ Not detected.

## Data Availability

Data are contained within the article.
